# Does Positive Airway Pressure Therapy Result in Improved Sleep Quality?

**DOI:** 10.4236/health.2014.618278

**Published:** 2014-10

**Authors:** Carl Stepnowsky, Tania Zamora, Christine Edwards

**Affiliations:** 1 Health Services Research & Development Unit, Veterans Affairs San Diego Healthcare System, San Diego, USA; 2 Department of Medicine, University of California, San Diego, USA

**Keywords:** Measurement, Continuous Positive Airway Pressure, Sleep Apnea Syndromes, Sleep Quality, Treatment Adherence

## Abstract

**Introduction:**

Positive airway pressure (PAP) therapy is the gold-standard for obstructive sleep apnea (OSA) management. While it is known that PAP is efficacious for controlling breathing events during sleep when it is worn at the right pressure for the amount of time prescribed, there is less clear data on how well it improves sleep quality. There are few studies that have examined the effectiveness of PAP therapy on sleep quality.

**Methods:**

OSA participants (*n* = 241) from a larger trial examining a PAP adherence were included. Participants were provided with PAP instruction and followed at 2 months and 4 months. PAP adherence was measured as the number of hours per night at prescribed pressure, an objective measure of treatment adherence. The Pittsburgh Sleep Quality Index (PSQI) was used as the primary measure of sleep quality.

**Results:**

The PSQI was significantly correlated with PAP adherence at both the 2-month and 4-month time points, such that lower sleep quality was associated with lower PAP use. This finding held for the sleep disturbance subscale of the PSQI. Over 55% of those using PAP therapy at the 4-month time point continued to report significantly disturbed sleep.

**Discussion:**

This study shows that PAP therapy does not appear to improve sleep quality to a degree that would be expected. Over half of those patients using PAP therapy still experienced disturbed sleep. Whether the disturbed sleep is directly attributable to the PAP device itself or to disturbed sleep secondary to uncontrolled OSA when PAP is not worn is worthy of further investigation.

## 1. Introduction

Obstructive sleep apnea (OSA) is a prevalent and serious chronic illness characterized by repeated complete or partial obstructions of the upper airway during sleep (apneas and hypopneas, respectively). OSA generally results in poor sleep quality, characterized by short sleep latency (*i.e.*, time to sleep onset), increased Stage 1 sleep, decreased rapid-eye movement (REM) and slow-wave sleep (SWS), poor sleep efficiency, and frequent sleep fragmentation caused by transient arousals [1] [2]. Poor sleep quality causes sleep to be non-restorative, resulting in mild to severe excessive daytime sleepiness (EDS). EDS and hypoxia secondary to OSA are associated with a number of neurocognitive, mood, and behavioral consequences, including lowered health-related quality of life (HRQOL) [3], impaired cognitive performance [4], impaired driving ability (2 to 7 times increased risk of a motor vehicle accident) [5], depressed mood [6], psychosocial disruption (e.g., more impaired work performance and productivity, and higher divorce rates) [7], and disrupted sleep and impaired quality of life of the spouses of OSA patients [8]. The goal of OSA treatment management is to normalize not just the breathing of OSA patients, but the sleep quality as well.

Positive airway pressure (PAP) therapy [9] is the gold-standard treatment for OSA [10], with meta-analytic reports showing improvement in daytime sleepiness [11] and HRQOL [12]. The standard prescription is to use PAP whenever asleep, including during daytime naps. However, despite PAP being the most efficacious treatment available to OSA patients, adherence is substandard (3 to 5 hours per night) [13].

The PAP device provides data on the number of “residual” apneas and hypopneas (*i.e*., those respiratory events measured while using PAP therapy). The residual apnea-hypopnea index (AHI) is defined as the number of apneas and hypopneas during PAP use time. Residual AHI provides a proxy measure of treatment efficacy to help determine the need for clinical management changes (e.g., pressure setting changes). However, there are several key issues concerning the efficacy data provided by the PAP devices. First, historically, residual AHI has not been able to distinguish obstructive from central events. Second, while PAP devices appear to accurately measure apneas, the number of hypopneas is often inaccurate relative to measures derived from standard sleep testing [14]–[16]. The tendency of PAP devices to over score hypopnea events can result in “over-management” by providers and excessive patient concern. Third, PAP devices measure breathing events and not sleep quality. It is well known that the AHI, which is the gold-standard measure of OSA disease severity, at best only moderately correlates with measures of OSA symptoms [17] [18]. While the residual AHI on PAP may be acceptably below 10 events per hour of sleep, the patient may still be experiencing OSA symptoms and suboptimal sleep quality. Finally, both AHI and sleep quality are unmeasured on that portion of the night that patients do not wear PAP, which is often the second half of the night when sleep breathing disturbances are worse. An evolving literature on PAP withdrawal studies shows that OSA and its negative effects resume when PAP therapy is not worn [19]-[21].

Only a few studies in the literature specifically examine the extent of poor sleep disturbance when PAP therapy is used. Wickwire and colleagues retrospectively examined medical charts that included a three-item clinic-developed insomnia scale, and found that 37% of the patients (*n* = 232) had at least one insomnia complaint while using PAP therapy [22]. This study showed that difficulty maintaining sleep was significantly associated with PAP adherence in this study (*p* > 0.05). Another study that employed data mining methods to examine the relationship between insomnia complaints (using the Insomnia Severity Index, or ISI) and PAP adherence found a moderate effect size of 0.43 (d-index), such that the low ISI group (*i.e*., low insomnia) used PAP more than the high ISI group (*i.e*., high insomnia) (4.4 ± 2.4 vs. 3.4 ± 2.2 hours per night) [23]. This study showed that insomnia symptoms were found in ~50% of patients with moderate-severe OSA [23]. Another study found that even those patients who used PAP ≥ 4 hrs per night had significant insomnia symptoms based on the Regensburg Insomnia Scale [24].

We had the opportunity to investigate the relationship between measures of sleep quality (using the Pittsburgh Sleep Quality Index) in new patients using PAP therapy over the first four months of PAP use.

## 2. Methods

The participants included in this study were involved with a larger clinical trial whose goal was to evaluate the effect of an intervention designed to increase adherence with PAP therapy. The design was a randomized parallel group trial with blinded evaluation that compared an Internet intervention based on the wireless telemonitoring of PAP data that fostered patient-centered collaborative care between patient and provider (*i.e*., patient-centered collaborative care for OSA, or **PC3**) versus a usual care PAP treatment protocol (*i.e*., Usual Care, or **UC**). Participants completed baseline, 2-month and 4-month assessments. The project took place over a 3-year period. UC was comprised of predetermined clinical contacts, while PC3 was comprised of as-needed clinical contacts, based on objectively measured PAP adherence and efficacy data and access to a patient-oriented website. Participants underwent identical instruction and education on OSA and PAP therapy and used identical PAP units. The study was designed as a practical clinical trial that compared one clinical care method against another, with the goal of informing clinical decision-making [25]. The effect of clinical care methods on a behavioral outcome (*i.e*., PAP adherence) was compared. Thus, the study was considered in large part a behavioral trial. Further details can be found in a previous publication [26].

### 2.1. Participants

The sample included 241 participants (82 women; 159 men) recruited and screened for suspicion of OSA by physicians in the University of California, San Diego (UCSD) Healthcare System Sleep Medicine Center. Inclusion criteria were a diagnosis of OSA (apnea-hypopnea index ≥ 15) [27], PAP therapy prescription, and age ≥ 18 years. Exclusion criteria included residence in a geographical area outside of San Diego County (which could make regular contact and participation difficult); fatal comorbidity (life expectancy less than 6 months, as indicated by treating physician); or significant documented substance/chemical abuse. All participants signed informed consent, and the study was approved by the UCSD IRB. The participants were offered financial reimbursement for participating and completing the study, and to modestly offset travel-related expenses.

Of the 241 participants were enrolled over the project period (115 to Usual Care and 126 to the PC3 group), the total number of withdrawals during the course of the project was 7. These were due to PAP intolerance or subsequent self-withdrawal from the study. Baseline rates of OSA patients with PAP intolerance or refusal are estimated to be about 20% in clinical practice. In our project, this worked out to be about 3%, significantly lower than base rates.

Because men have a greater risk for sleep apnea (and are identified and diagnosed at a greater rate) than women, a 4:1 ratio (men:women) was expected. With 82 women and 159 men enrolled, the actual men:women ratio was closer to 2:1. The expected percentage of women was 20% while the actual percentage was just over 33%. Minorities were recruited approximately in the percentages expected (expected vs. actual): American Indian/Alaska Native (1.5% vs. 1.7%); Asian (8.0% vs. 9.2%; Native Hawaiian vs. Other Pacific Islander (0.5% vs. 0.0%); and Black or African-American (5.0% vs. 3.3%); while Whites were 85% vs. 84.6%. Participants from other (or unclassified) races were not expected per original enrollment table) and the study enrolled three (or 1.3%) participants reported their race as “other”. Despite our geographical location, the study only enrolled 10% participants who self-reported as Hispanic. Table 1 shows the baseline characteristics of the total group and by intervention group. This analysis provides support that the randomization resulted in no significant differences between the intervention groups on age, weight, disease severity, or sleepiness level.

### 2.2. Apparatus

Participants in this study were provided with a Positive Airway Pressure device (PAP; Autoset II, Res Med, San Diego, CA). A wireless modem attached to their PAP device sent the data from the device to a Web-portal accessible by our team. The web-portal (“Restraxx Data Center,” or RDC) is comprised of the wireless module and the server/database that houses the data. The portal is fully compliant with the Health Insurance Portability and Accountability Act of 1996 (HIPAA), restricting access to use by authorized health care professionals. The wireless module connects to the flow generator via a docking mechanism that allows the connection to an existing 15-pin expansion port at the rear of the flow generator. All PAP devices were outfitted with a humidifier. Participants were given a choice between full face and nasal mask types and were given the opportunity prior to the study to wear the masks, experience the positive airway pressure, and work through any initial problems or issues.

### 2.3. Measures

Measures were assessed at baseline, 2 months, and 4 months and included participant sociodemographics, OSA symptoms, Pittsburgh Sleep Quality Index (total score and subscale scores), Epworth Sleepiness Scale (ESS), Sleep Apnea Quality of Life Index (SAQLI), and the Center for Epidemiologic Studies-Depression (CES-D) scale. Demographic information assessed included age, gender, education, marital status, height, and weight. The Apnea-Hypopnea Index (AHI), a count of the total number of apneas and hypopneas per hour of sleep, was measured via overnight sleep study.

PAP adherence is defined as the amount of time that the device is worn at the prescribed pressure. It is reported as the number of hours per night and as such, represents the cumulative total of hours of device use over a defined period of time (e.g., over 2 months and 4 months of time). It is important to note PAP adherence does not include the amount of time that the unit is powered on but not at prescribed pressure. Because of this, PAP adherence is an accurate objective measure of treatment adherence.

The Pittsburgh Sleep Quality Index (PSQI) is a self-reported scale aimed at assessing sleep quality and disturbances over a 1-month period [28] [29]. The PSQI measures 7 areas of sleep: subjective sleep quality, sleep latency, sleep duration, habitual sleep efficiency, sleep disturbances, use of sleep medication, and daytime dysfunction [28]. The PSQI total score of greater than 5 is indicative of poor sleep quality, while a score of 5 or less is indicative of good sleep quality [30]. Of particular relevance to this paper are two PSQI subscales: 1) Sleep Quality, which is comprised of 1 item that asks, “During the past month, how would you rate your sleep quality overall?” The 1-item Sleep Quality question is based on 0 = very good; 1 = fairly good; 2 = fairly bad; 3 = very bad. 2) Sleep Disturbance is comprised of 9items that asks, “During the past month, how often have you had trouble sleeping because you···?” This PSQI subscale results in one of four categories, anchored by 0 = better and 3 = worse.

The Epworth Sleepiness Scale (ESS) is an 8-item validated measure of daytime sleepiness [31]. It asks respondents to estimate how likely they are to doze in 8 different situations. The ESS is able to discriminate the sleepiness level of OSA patients from that of normal controls [31]. The score is based on a 0 – 24 point scale, with higher scores representing greater levels of sleepiness. Self-rated sleepiness was also assessed via a modified Visual Analog Scale (VAS). The score was the number circled on a range from 0 to 10, with the higher score indicating more sleepiness.

Depressive symptoms were measured using the Center for Epidemiological Studies-Depression Scale short form (CES-D). The CES-D is a 10-item self-report measure of depression [32]. The 10-item version has adequate predictive accuracy when compared to the original full-length 20-item version, as well as adequate test-retest correlations and discriminative validity [33].

### 2.4. Data Analysis

Means and standard deviations were calculated for the variables of interest. Paired sample t-tests were used to test mean difference between the groups or indices. Pearson correlation coefficient was used for continuous data. Data were analyzed using SPSS v 17.0 (Chicago, IL). Given that the only differences between the intervention and control groups was frequency of contact, and there were no differences between the groups on the type of PAP device used or kind of clinical support, some results are reported for the total group.

## 3. Results

Table 1 shows the baseline characteristics of the total group, which was middle-aged, overweight, sleepy, and had an average moderate-severe sleep apnea severity level. Thirty-four percent of the sample were women (81 women, 159 men). Race breakdown was as follows: American Indian/Alaska Native (1.7%), Asian (9.2%), Native Hawaiian/Other Pacific Islander (0.0%); Black/African-American (3.3%), White/Caucasian (84.6%), and “other” (1.3%).

**Figure 1.**
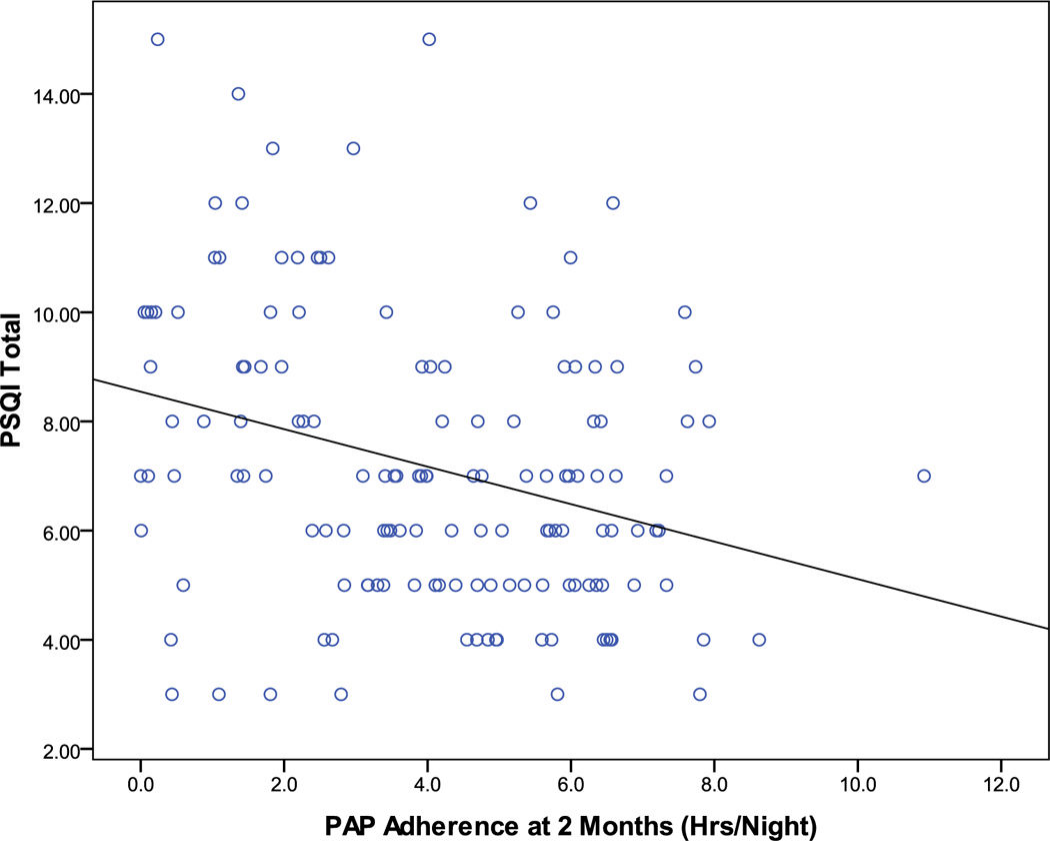
Scatterplot of PAP adherence by PSQI total score at the 2-month time point. The plot shows the line of best fit.

Table 2 shows the Pittsburgh Sleep Quality Index scores by intervention group across time (baseline, 2 months, and 4 months). The intervention groups did not differ on PSQI Total Score or on any PSQI subscale scores. The Pearson correlation coefficient between PAP adherence and PSQI Total Score, Sleep Quality subscale, and Sleep Disturbance subscale at 2 months was −0.303 (*p* < 0.0001), −0.131 (*p* = 0.065) and −0.181 (*p* = 0.01) and at 4 months was −0.301 (*p* < 0.0001), −0.035 (NS) and −0.217 (*p* = 0.003). Figure 1 shows the scatterplot between PAP adherence and PSQI Total Score at the 2-month time point. Finally, PSQI scoring guidance recommends that a threshold of 5 be used for the PSQI. A PSQI Total Score greater than or equal to 5 indicates poor sleep quality. Given that the mean PSQI Total Score across all time points is greater than 5, this sample’s sleep quality can be characterized as poor.

**Figure 2.**
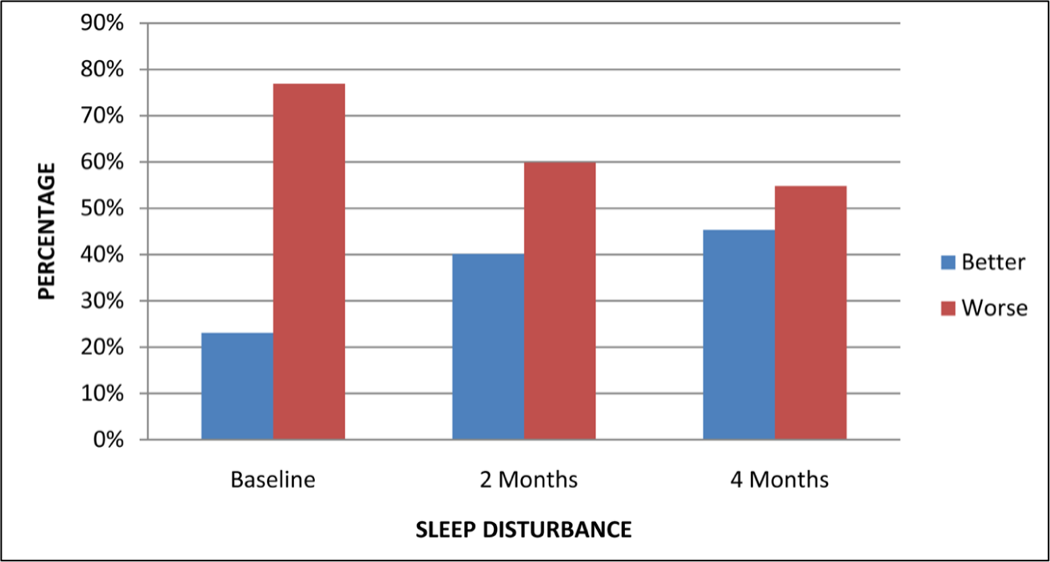
PSQI sleep disturbance subscale score at baseline, 2 months, and 4 months.

On the PSQI Sleep Disturbance subscale, none of the 240 participants had a Sleep Disturbance score in the lowest (*i.e*., least disturbed or score = 0) group, indicating that all had some level of disturbed sleep. At baseline, 23.1% were in the lowest half of sleep disturbance (*i.e*., score = 0 or 1). At 2 months and 4 months, this percentage increased to 40.1% and 45.3%, respectively. This means that 59.9% and 54.7% of the group reported having disturbed sleep while on PAP therapy at the 2-month and 4-month timepoints, respectively. Figure 2 shows the PSQI Sleep Disturbance subscale scores over the three time points.

When the Sleep Disturbance subscale was classified into two groups (high and low), PAP adherence differed by group at both the 2-month and 4-month time points, such that PAP adherence was higher at both time points for the participants with less disturbed sleep (4.5 ± 2.0 vs 3.8 ± 2.3 hrs/nt; *p* = 0.02 and 4.7 ± 2.1 vs 3.8 ± 2.1 hrs/nt; *p* = 0.004), respectively.

Based on the total group of OSA patients at baseline, 71.6% rated their sleep as “very good” on the PSQI Sleep Quality subscale. While on PAP therapy at the 2-month time point, however, that percentage decreased to 62.2%. Stated differently, at baseline 28.4% of those diagnosed with OSA rated their sleep as less than “very good,” but then while on PAP therapy 2 months later, the percentage increased to 37.8%. The same percentage held at the 4-month time point (~38% rated their sleep as less than “very good”).

The mean PAP adherence differed between the two groups at the 2-month time point (4.2 ± 2.3 vs. 3.5 ± 2.3 hrs/nt; *p* = 0.04) but not at the 4-month time point (4.4 ± 2.4 vs 3.9 ± 2.3 hrs/nt; *p* = 0.14). The overall mean adherence for the total group at the 2-month and 4-month time points were 3.9 ± 2.3 and 4.2 ± 2.4 hrs/nt, respectively. Generally speaking, this level of PAP adherence represents an acceptable level of use. The commonly accepted goal of clinical management with PAP therapy is to use it more than 4 hrs/nt on more than 70% of nights (per Centers for Medicare and Medicaid Services (CMS) reimbursement guidelines). This standard results in a minimal mean adherence of approximately 2.8 hrs/nt (70% × 7 nights × 4 hrs/nt). Our study mean adherence of approximately 4 hrs/nt is over 40% higher than that of the CMS standard.

## 4. Discussion

OSA is the most common of the sleep-related breathing disorders (SRBD). For the SRBDs, standard clinical management is to treat the nocturnal breathing disorder, with the assumption that sleep will be improved with the resolution or control of the breathing disorder. There is an evolving school of thought that the level of sleep disturbance in OSA patients who have been prescribed and regularly use PAP therapy may be underappreciated. This idea is supported by the result of the current study, which found that a large sample of OSA patients who were using PAP therapy with an overall acceptable level of use continued to report disturbed sleep 2 months and 4 months after starting therapy.

**Figure 3.**
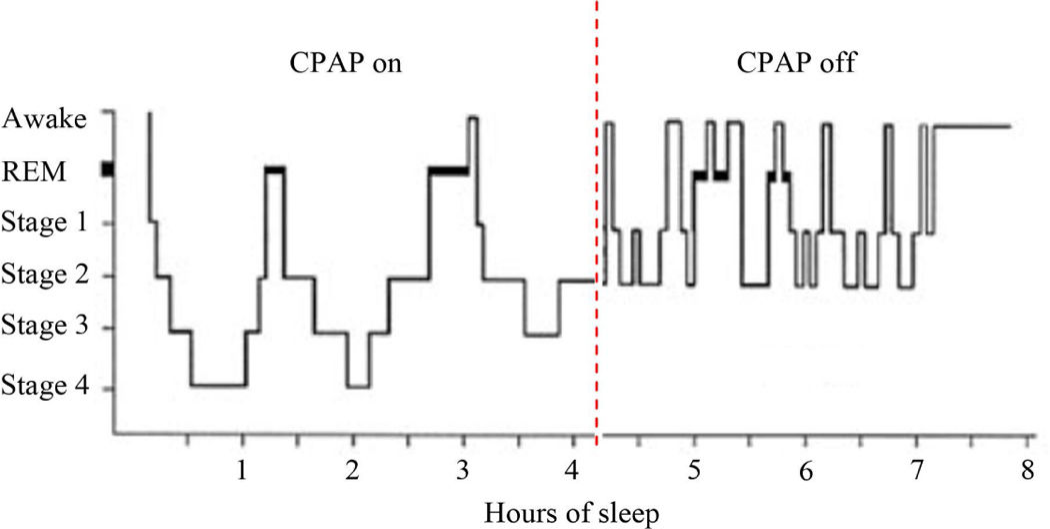
Partial PAP use pattern hypnogram based on hypothetical data. The first half of the night assumes ideal conditions, including use of the correct pressure setting, appropriate mask fit (and acceptable mask leak), and ideal use by the patient. The second half of the night assumes resumption of OSA and its consequences on sleep architecture.

Sleep architecture is an important driver of perceived sleep quality. While it is expected that sleep architecture would be normalized by PAP therapy, there are some findings that this may not be the case for all users [19] [20]. There are several possible reasons for PAP’s inability to fully normalize sleep: 1) PAP therapy is typically not used across the sleep period, so OSA can cause disruptions to sleep when therapy is not being used, and 2) even when PAP is used, it may be that the therapy itself is causing disruptions to sleep. PAP can cause disruptions to sleep for a number of reasons, including pressure intolerance, mask discomfort, and suboptimal control of OSA due to incorrect pressure settings, among others. Figure 3 provides a hypothetical hypnogram showing normal sleep in the first half of the night and the hypnogram from a typical OSA patient in the second half of the night. This hypnogram shows relatively normal sleep architecture on the first 4 hours of the night when PAP is worn. When PAP is removed in the middle of the night, breathing disturbances may resume, and sleep is once again disrupted.

Another evolving line of research concerns the relationship between sleep apnea, depression, fatigue, and sleep quality. Research has shown that depressive symptoms are more significantly associated with daytime fatigue than with objective measures of OSA severity [34] [35]. Other research has shown that depressive symptoms are associated with poor sleep quality [36]. Sleep quality is of increasing interest in those with depressive symptoms, because antidepressant trials have shown that depressive symptoms and self-reported sleep quality can both improve, even while polysomnographic measures of sleep quality worsen [37] [38]. More recently, it was shown that self-reported sleep quality is independently associated with daytime fatigue, even after taking into account demographics, comorbid conditions, OSA disease severity, and depressive symptoms [39]. This finding emphasizes the importance of closely following sleep quality in the clinical management of OSA.

The present study does have limitations. Measurement of sleep quality is achieved by self-report methods and does not include an objective measure of sleep/wake patterns or of sleep architecture. Future studies would do well to include an objective measure of sleep quality.

In the current study those who reported less disturbed sleep had significantly better PAP adherence. This study can only speculate about the reasons why this was the case. Future studies will need to explore the role of other important factors, such as recurring OSA during that portion of the night that therapy was not worn, and other sleep-related issues, such as insomnia.

## Figures and Tables

**Table 1.** Baseline characteristics by and across groups (81 women and 159 men).

	Both groups	PC3 (*N* = 126)	Usual care (*N* = 114)	Range (*N* = 240)

	*Mean ± SD*	*Mean ± SD*	*Mean ± SD*	
Age	52.1 ± 13.3	52.2 ± 13.0	51.9 ± 13.6	19–85
Body mass index (kg/m^2^) (BMI)	32.4 ± 8.0	32.1 ± 8.3	32.8 ± 7.8	20–64
Apnea-hypopnea index (AHI)	36.5 ± 25.9	36.3 ± 24.9	36.6 ± 27.0	7–126
Epworth sleepiness scale (ESS)	10.6 ± 5.3	10.7 ± 5.2	10.5 ± 5.4	1–24
Center for epidemiological studies-depression (CESD)	11.2 ± 5.5	11.3 ± 5.2	11.0 ± 5.9	2–28

**Table 2.** Pittsburgh Sleep Quality Index (PSQI) by intervention group over time.

Subscale	Total group	Baseline			Total group	2-month visit			Total group	4-month visit		

		UC	PC3	*p*-value		UC	PC3	*p*-value		UC	PC3	*p*-value
PSQI total	9.6 ± 2.5	9.6 ± 2.6	9.6 ± 2.3	0.89	7.2 ± 2.6	7.0 ± 2.8	7.3 ± 2.5	0.40	6.2 ± 2.5	6.0 ± 2.7	6.5 ± 2.4	0.26
Sleep quality	0.61 ± 1.1	0.58 ± 1.1	0.64 ± 1.1	0.68	0.5 ± 1.0	0.5 ± 0.98	0.58 ± 1.0	0.47	0.5 ± 1.0	0.5 ± 1.0	0.5 ± 1.0	0.87
Sleep disturbance	2.0 ± 0.70	1.9 ± 0.68	2.0 ± 0.70	0.40	1.7 ± 0.61	1.6 ± 0.60	1.7 ± 0.61	0.45	1.6 ± 0.61	1.6 ± 0.62	1.6 ± 0.61	0.49
Sleep latency	1.0 ± 0.97	1.0 ± 0.94	0.9 ± 1.00	0.72	0.9 ± 0.95	0.9 ± 0.89	0.89 ± 1.0	0.87	0.8 ± 0.87	0.8 ± 0.93	0.8 ± 0.83	0.83
Sleep duration	3.0 ± 0.0	3.0 ± 0.0	3.0 ± 0.0		2.5 ± 0.58	2.5 ± 0.56	2.8 ± 0.60	0.84	1.8 ± 0.62	1.8 ± 0.66	1.8 ± 0.59	0.69
Daytime dysfunction	2.1 ± 0.79	2.1 ± 0.80	2.2 ± 0.78	0.65	1.4 ± 0.79	1.3 ± 0.83	1.4 ± 0.75	0.12	1.3 ± 0.83	1.2 ± 0.84	1.4 ± 0.81	0.17
Use of sleep medication	0.65 ± 0.96	0.64 ± 0.96	0.66 ± 0.96	0.87	0.3 ± 0.58	0.2 ± 0.64	0.3 ± 0.53	0.92	0.3 ± 0.68	0.2 ± 0.57	0.4 ± 0.75	0.05

Note: PC3 = patient-centered collaborative care; PSQI = Pittsburgh Sleep Quality Index; UC = usual care.
